# Optimizing setup of scan number in FTIR spectroscopy using the moment distance index and PLS regression: application to soil spectroscopy

**DOI:** 10.1038/s41598-021-92858-w

**Published:** 2021-06-25

**Authors:** Issam Barra, Lotfi Khiari, Stephan M. Haefele, Ruben Sakrabani, Fassil Kebede

**Affiliations:** 1 Center of Excellence in Soil and Fertilizer Research in Africa (CESFRA), AgroBioSciences, Mohammed VI Polytechnic University (UM6P), 43150 Benguerir, Morocco; 2 Department of Soils and Food Engineering, Faculty of Agriculture and Food Sciences, Laval University, Quebec, Canada; 3 Department of Sustainable Agriculture Sciences, Rothamsted Research, Harpenden, UK; 4 School of Water, Energy and Environment, Cranfield University, Cranfield, UK

**Keywords:** Analytical chemistry, Cheminformatics, Green chemistry

## Abstract

Vibrational spectroscopy such as Fourier-transform infrared (FTIR), has been used successfully for soil diagnosis owing to its low cost, minimal sample preparation, non-destructive nature, and reliable results. This study aimed at optimizing one of the essential settings during the acquisition of FTIR spectra (viz. Scans number) using the standardized moment distance index (SMDI) as a metric that could trap the fine points of the curve and extract optimal spectral fingerprints of the sample. Furthermore, it can be used successfully to assess the spectra resemblance. The study revealed that beyond 50 scans the similarity of the acquisitions has been remarkably improved. Subsequently, the effect of the number of scans on the predictive ability of partial least squares regression models for the estimation of five selected soil properties (i.e., soil pH in water, soil organic carbon, total nitrogen, cation exchange capacity and Olsen phosphorus) was assessed, and the results showed a general tendency in improving the correlation coefficient (R^2^) as the number of scans increased from 10 to 80. In contrast, the cross-validation error RMSECV decreased with increasing scan number, reflecting an improvement of the predictive quality of the calibration models with an increasing number of scans.

## Introduction

Since the middle of the last century, Nelson et al.^1^ have shown good evidence that the rational use of agricultural soil analysis can contribute to better soil management. Soil analysis was initiated under the impetus of Bray^2^ in the development of analytical procedures to quantify soil nutrient reserves. A few years later (1956), Fitts and Nelson^3^ proposed to use soil analysis to suggest fertilization and liming practices, to predict the probability of economic response to fertilizers, to assess soil productivity and to improve soil productivity through amendments or cropping practices.

Soil diagnosis is a very important task allowing the knowledge of its nature such as the particle size distribution, acidity, status of nutrient availability and others, which influence the soil productivity thereby controlling crop productivity^4^. However, conventional soil analysis with a range of chemical methods is slow, labor intensive and expensive. But recent developments promise to greatly simplify soil diagnosis, to make it faster, cheaper and more suitable for routine analysis. This effort of simplification continues and is mostly based on the advent of dry chemistry applied especially to spectroscopy.

Over the last decades, infrared spectroscopy techniques have been used increasingly not only for identifying molecular bands, but as rapid diagnostic tools. Methods based on the absorbance/reflectance of infrared emissions offer several advantages compared with conventional agrochemical ones, and soil spectroscopy has shown to be a fast, cost-effective, environmentally friendly, non-destructive, reproducible and repeatable analytical technique. Currently, the use of these technique has become a trend, especially with the encouragement of green chemistry tools for assessing various soil physical, chemical and biological properties^5^.

The advances in instrumentation, i.e. the development of fast, low cost, reproducible and portable instruments available for infrared techniques (medium and near infrared) have opened new opportunities for researchers to benefit of their capabilities, especially when combined with multivariate calibrations. The latter have shown to be powerful tools to develop quantitative and qualitative models in many fields including soil^6–10^, food^11,12^ pharmaceutics^13^ and petroleum^14–18^ analysis.

The high-sensitivity infrared spectral techniques will in all cases produce a spectrum, but the quality (stability, repeatability, reproducibility, noise, etc.) of this acquisition can vary dependent on the operation settings. This is why care should be taken of the very fine tuning of its parameters, namely the resolution (the recording step of the spectra) and the scan number per sample which allows averaging several acquisitions in order to reduce the noise of measurement by recording the same signal repeatedly. Working with the optimal setup will undoubtedly improve the measurements stability and repeatability^19,20^.

Almost in all research methodologies of infrared spectroscopic studies, the setting of scans number leading to an averaged spectra is done in a non-scientific manner, based on either experience of the operator or on the instrument supplier’s general recommendation^21–24^. To improve this approach, the determination of the optimal number of scans requires the evaluation of the spectral stability. This can be done by the calculation of several metrics, viz., standard deviation of absorbencies of the MIR range, i.e., 4000–400 cm^−1^ ^25^, moment distance index (MDI)^26^, and more.

To address this issue, we hypothesized that the use of the moment distance index (MDI) as a metric could provide valuable information on the similarity between repeated spectra taken on the same soil sample and under the same settings. This MDI should guarantee a stable spectral signature and subsequently a consistency in the prediction of the physico-chemical properties of soil samples^27^. The objective of this work is to support efforts to establish quality control standards for spectral analysis using MDI to define (i) the optimal number of scans per replica, (ii) the number of replicates sufficient to obtain the best spectral stability and (iii) to evaluate the effect of variating the number of scans on soil property prediction.

## Materials and methods

### Soil samples and FTIR spectra acquisitions

**Figure 1:**
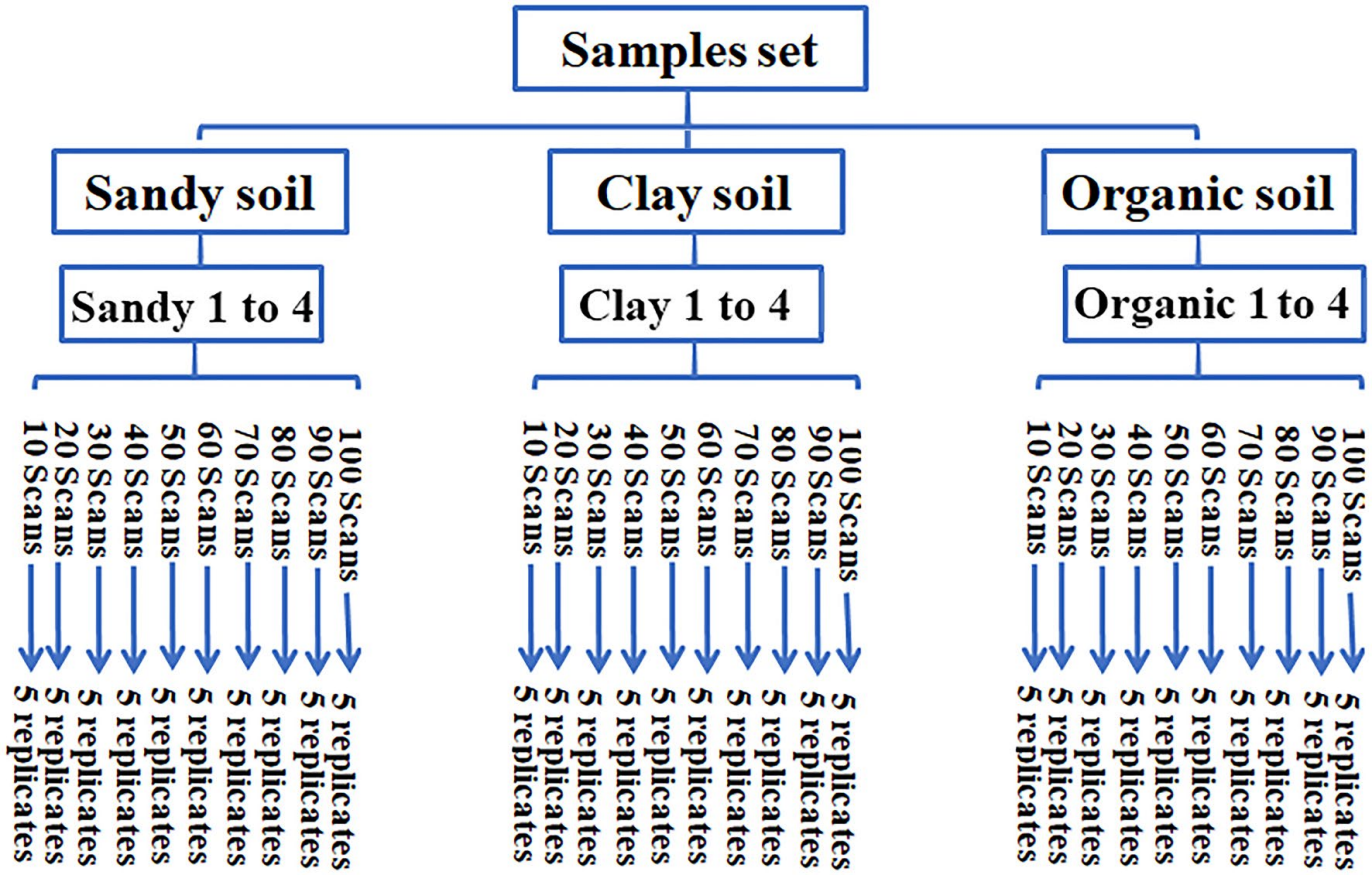
Scheme of the workflow for building the database used for the optimization of the number of scans/replica in FTIR analysis of the twelve reference soil samples.

Twelve oven-dried (at 39 °C for 48 h) and finely ground reference soil samples from the Wageningen Evaluating Programs for Analytical Laboratories (WEPAL) Netherlands, representing three types of soils (i.e., sandy, clay and organic) were used for the optimization of the scan number setup. The spectra recording was conducted between 4000 and 600 cm^−1^ on a Bruker Tensor II bench-top spectrometer at the Soil Spectroscopy Laboratory (CESFRA) of the Mohammed VI Polytechnique University, Morocco. The resolution was 4 cm^−1^ and for each sample 50 spectra were recorded. The setup variable was the number of scans that would be averaged to get the final spectrum at 10, 20, 30, 40, 50, 60, 70, 80, 90 and 100 scans, and five replications were recorded for each number acquisition (Fig. 1). In a second experiment, 40 soil samples representing different Moroccan regions were used to assess the effect of the scan number on the accuracy of predictive models. The samples were conditioned in plastic flasks and stored in a desiccator cabinet from Nalge company (New York USA). All samples were finely ground and dried at 39 °C for 48 h before FTIR spectra collection, using again the Bruker Tensor II bench-top spectrometer between 4000 and 600 cm^−1^ with different number of scans per measurement, i.e., 10, 20, 40, 60 and 80.

### Soil property measurements

Soil pH (1/5 in water), total organic carbon (TOC) (Sulfochromic oxidation chemical method), total nitrogen (TN) Kjeldahl method, cation exchange capacity (CEC) hexamine-cobalt method, and available phosphorus (Olsen method) were analyzed in the Soil Testing Laboratory of the Agricultural Innovation and Technology Transfer Center (AITTC-UM6P) following routine procedures as described in ISO 10390, ISO 14235, ISO 11261, NF ISO 23470, and ISO 11263, respectively.

### Moment distance index computation

**Figure 2:**
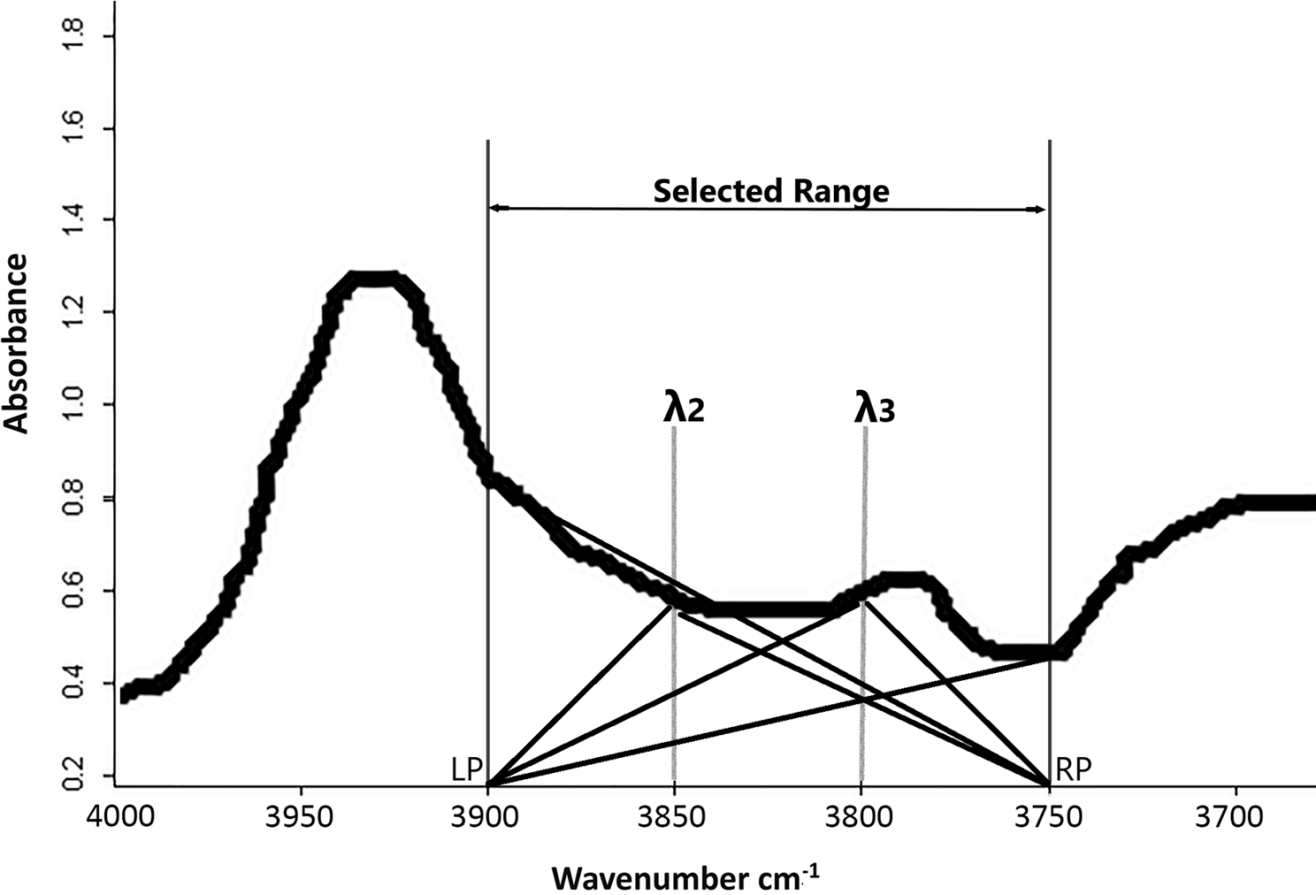
Schematic diagram for MDI calculation for spectral reflectance curve (the number of points between LP and RP pivots can vary depending on the spectral resolution and the width of the selected range)^26^.

The Moment Distance (MD) is a matrix of distances computed from two reference locations (pivots) to each spectral point within the selected range. Suppose a reflectance curve is displayed in Cartesian coordinates with the abscissa displaying the wavelength ‘λ’ and the ordinate displaying the reflectance ‘ρ’ (Fig. 2); the subscript ‘LP’ refers to the left pivot (shorter wavelength) and ‘RP’ designates the right pivot (longer wavelength). Let λ_LP_ and λ_RP_ are the wavelength locations observed at the left and right pivots for a reflectance data, respectively, where left (right) indicates a shorter (longer) wavelength. The proposed MD approach can be described in a set of equations^26^.

1 $$ MD_{{LP}}  = \mathop \sum \limits_{{i = \uplambda _{{{\text{LP}}}} }}^{{\uplambda _{{{\text{RP}}}} }} \left( {\uprho _{i}^{2}  + \left( {i - \uplambda _{{{\text{LP}}}} } \right)^{2} } \right)^{{0.5}} $$

2 $$ MD_{{RP}}  = \mathop \sum \limits_{{i = \uplambda _{{{\text{RP}}}} }}^{{\uplambda _{{{\text{LP}}}} }} \left( {\uprho _{i}^{2}  + \left( {\uplambda _{{{\text{RP}}}}  - i} \right)^{2} } \right)^{{0.5}} $$

3 $$ MDI = MD_{{RP}}  - MD_{{LP}} $$

4 $$ SMDI = \frac{{MDI - min\left( {MDI} \right)}}{{max\left( {MDI} \right) - min\left( {MDI} \right)}} $$

According to Salas et al.^26^, the first step is the calculation of the MD_LP_ and the MD_RP_ representing successively the moment distance from the left pivot and moment distance from the right pivot by applying the Eqs. (1) and (2). Then, the moment distance index of the selected part of the spectrum is the difference between the computed values of MD_RP_ and MD_LP_ Eq. (3).

SMDI is the standardized value of calculated MDIs with min(MDI) is the minimal MDI value calculated for the sample and max(MDI) is the maximal value Eq. (4). The standardized values of the index are calculated to make it easy when graphically representing the MDIs with a simple scale between 0 and 1.

### Chemometrics analysis

Chemometrics is a part of analytical chemistry that uses mathematical modeling and computer tools to extract important information from analytical data^28^. It is used to reduce data dimension and investigate the relationships between samples and variables^12,29^.

Chemometrics tools are divided into two main categories, namely, unsupervised methods (i.e., Principal Components Analysis), which are used as exploratory methods and supervised methods, that are used for predictive purposes^30,31^.

Partial Least Square (PLS) regression is a supervised method and very widely used with spectroscopic data^32^. PLS is the standard chemometric tool applied to perform calibrations and predictions. It models the relationship between two matrices, **X** (spectroscopic data) and **Y** (variable to be predicted) by finding linear combinations of **X** and **Y** matrices that are called latent variables (LVs)^33^.

In this study, the predictive models were built using the entire FTIR spectra measured on the soil samples (**X** matrix). In order to improve the predictive ability of the models, the 1st derivative preprocessing was applied^34^. It is the simplest form of Savitzky-Golay derivatives in which each variable, corresponding to a given wavelength, is subtracted from its immediate neighboring variable to eliminate the common part of the signal. The “leave one out” cross validation method^35^ was used as validation tool, which made it possible to calculate the figure of merit (R^2^ and RMSECV) required to test the predictive quality of the PLS models.

### Statistical criteria for assessing the quality of the PLSR models

To evaluate the performance of the PLS models, several figures of merit were tested including the cross-validation error or root mean squared error of cross-validation (RMSECV), and the correlation coefficient R^2^ ^36,37^.

### Software and data processing

The calculations of the SMDIs were performed using free and open-source software R-packages from the R Foundation for Statistical Computing whereas the set-up of the PLS models was done on OPUS Quant II 8.1 software from Bruker Optiks GmbH. The plotting of Figs. 5 and 6 was done using the Excel software from Microsoft 365.

## Results and discussion

### FTIR spectra

**Figure 3:**
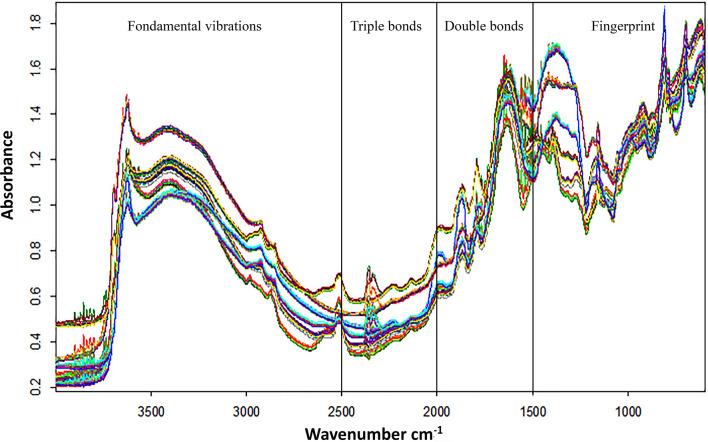
FTIR spectra of the twelve reference soil samples (50 replicates/sample) in the Mid-infrared range (600 to 4000 cm^−1^).

As shown in Fig. 3, the Mid-infrared spectra of soil samples can be divided into four parts, from 4000 to 2500 cm^−1^ which represents the fundamental vibrations generally caused by O–H, C–H, and N–H stretching, the triple bonds stretching vibrations from 2500 to 2000 cm^−1^, the region between 2000 and 1500 cm^−1^ covering the double bonds vibrations, and the range between 1500 to 400 cm^−1^ representing the fingerprint^38^. Given the complexity of the soil matrix, the spectra show several absorbances representing the different types of chemical bonds. The peaks around (3800–3600 cm^−1^) are linked to O–H stretching in clay minerals^39^. The spectral signatures near to 3550 cm^−1^ are associated to the Al–OH vibrations which come from kaolinite^40^. The bonds around 2500 cm^−1^ can be assigned to carbonate (calcite)^41^. The nitrile group (C–N) can be observed between 2200 and 2300 cm^−1^, and the principal bands in the 1500–2500 cm^−1^ region are ascribed to C=C and C=O stretching^38^. The interpretation of peaks in the region below 1000 cm^−1^ is difficult since it characterizes the fingerprint of the mineral compounds^39^.

### Scan number optimization using the standardized moment distance index

**Figure 4:**
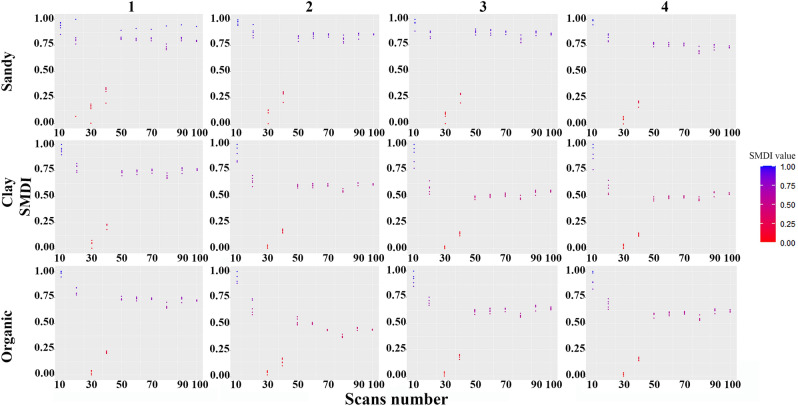
Calculated standardized moment distance index (SMDI) for all spectra of the twelve soils samples resulting from different scan numbers. The points’ (SMDI) rapprochement indicates the improvement of the spectra’s similarity.

The standardized moment distance index was used in this study as a metric that could detect the fine changes of the curve and extract the maximum similarity between the spectra of the same sample using the same acquisition setup (i.e. number of scans), and expressing the result in a single value for the whole spectrum. The obtained individual SMDI values are not of importance but rather their variation for the repetitions, as the target is to find a constancy of the SMDI values for the spectra taken with the same setting (scans number). The results (Fig. 4) showed that, on the one hand, the lower the number of scans, the more distance between repetitions is noticed. More precisely, below 50 scans the difference between the two extreme values of each setting is around 0.20 SMDI units, but above 50 scans this difference decreases continuously to about 0.02 SMDI units at 100 scans which represents the maximum number of scans conducted and which provides a very good recording stability. It can be concluded that compared to the reference value of SMDI at 100, the spectral similarity increases in all cases with more than 50 scans.

The twelve graphs in Fig. 4**,** representing the two contrasting soil types (organic and mineral) and the two extreme textures (sand and clay), all show the same conical pattern of the SMDI model distribution as a function of the number of scans. This lying funnel shape shows the largest opening on the left side and corresponds to the greatest disparity with respect to the reference SMDI of 100 scans. And they also all show a sill of convergence to the number of 100 scans to the right of the funnel. This funnel pattern for the 12 graphs shows a binary partition between a group of below 50 scans with widely dispersed SMDI values, and a group of above 50 scans with constant SMDI values approximating the SMDI values at 100 scans. When compared with similar studies in soil surveys using mid-infrared libraries, the 32 spectrum scans taken arbitrarily by Seybold et al.^42^ are not sufficient to converge to spectra stability. But the 60 scans acquired and averaged to produce a reflectance spectrum by Baldock et al.^43^ seem more adequate and stable when correlating to soil properties.

On the other hand, the present study was based on very contrasting soil types, viz. sandy soils, clay soils and organic soils to cover a wide variability to explore if these soil types behave the same or differently. But the results showed that the various soil types respond in the same way and the spectral similarity is always better beyond 50 scans.

With respect to the quality control standards, this study tested two essential criteria in the field of soil spectroscopy, namely the repeatability (5 levels of repetition of the same number of scans for the same sample) and the reproducibility (4 samples of each soil type). The results have shown that the tested criteria (viz., repeatability and reproducibility) become validated when the number of scans is more than 50. However, please note the outlier in the sandy soil 1, representing a repeatability problem which was probably due to the preheating conditions of the instrument, because this was the first sample to be analyzed during the experiment. This happened even though the preheating conditions required by the CESFRA Soil Spectroscopy Laboratory protocol (wait for 30 min after turning on the instrument) have been respected.

### Effect of the scan number on the precision of predictive models

**Figure 5:**
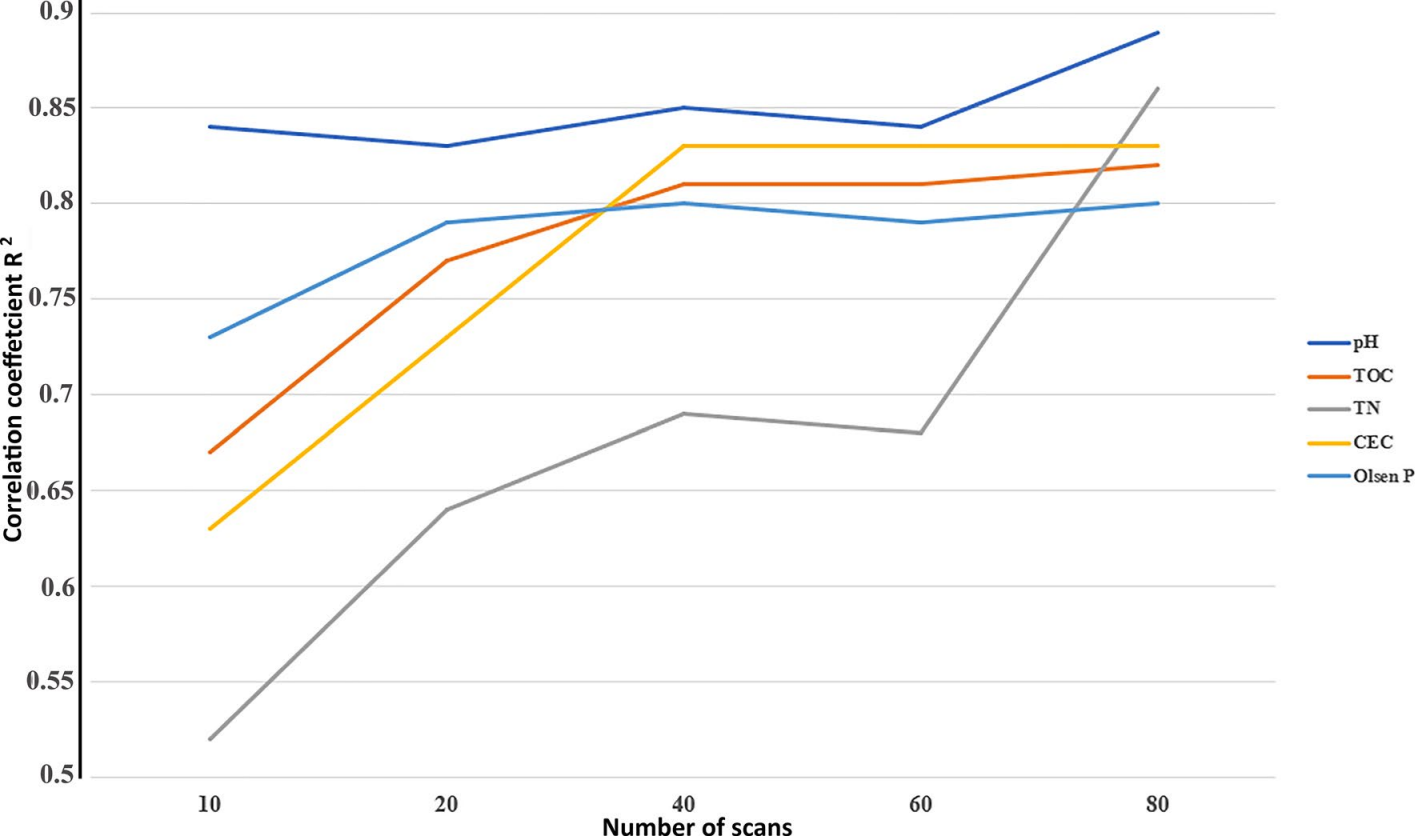
Variation of the correlation coefficients according to the number of scans fixed during the acquisition of the FTIR spectra of the forty soil samples.

**Figure 6:**
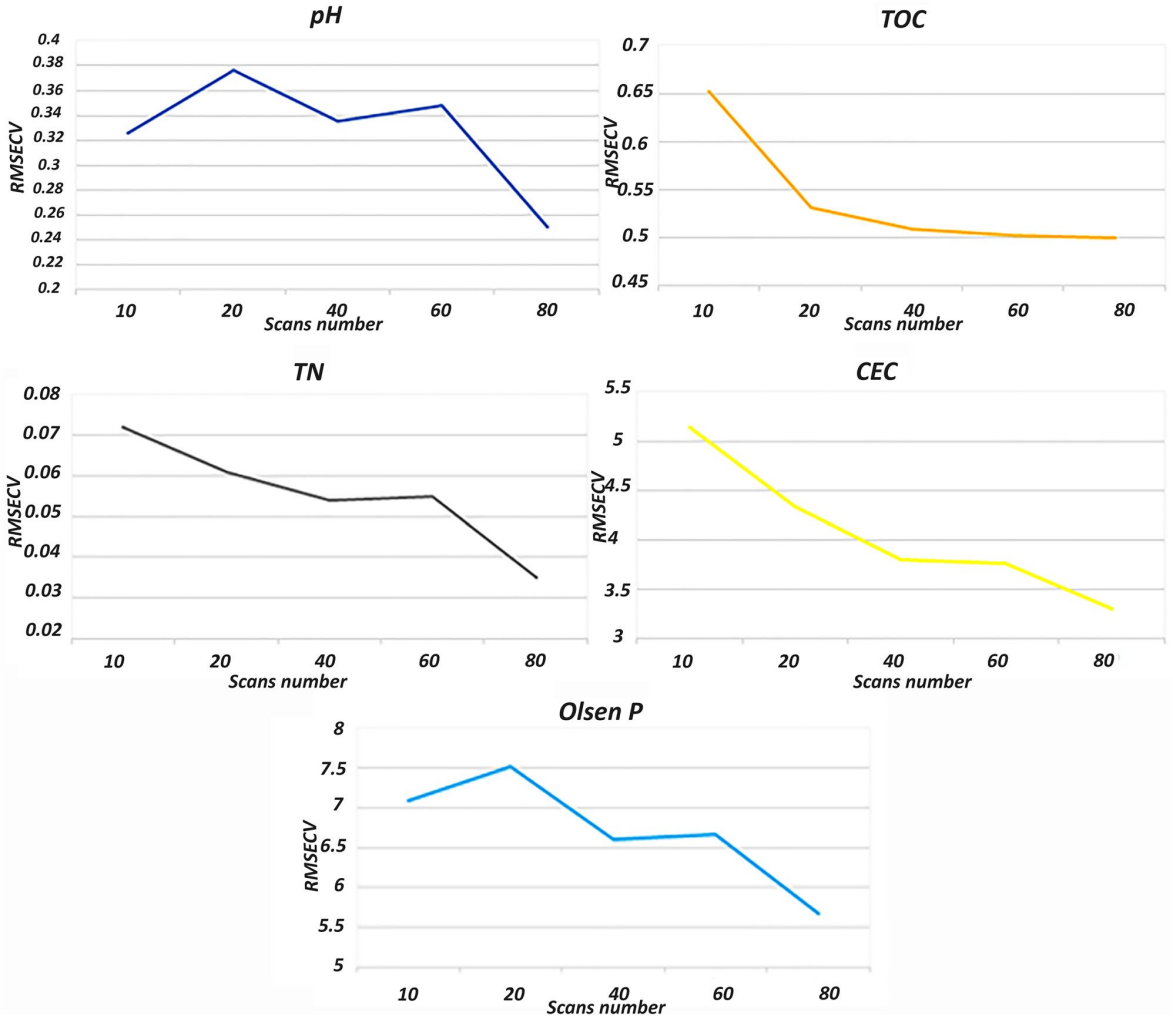
Variation of the root mean squared error of cross validation (RMSECV) for five selected soil properties according to the selected scan number setup of forty soil spectra acquisition.

To better highlight the effect of the scan number on the predictive models, partial least squares regression was used to set up five calibrations for each soil property against the scan number viz. 10, 20, 40, 60 and 80 scans using a set of forty soil samples. A general increasing trend in the correlation coefficient R^2^ was observed with the increasing scan number from 10 to 80 for all the regression models (Fig. 5), whereas the opposite was observed for the cross-validation error RMSECV which decreased with increasing scan number from 10 to 80 (Fig. 6).

**Figure 7:**
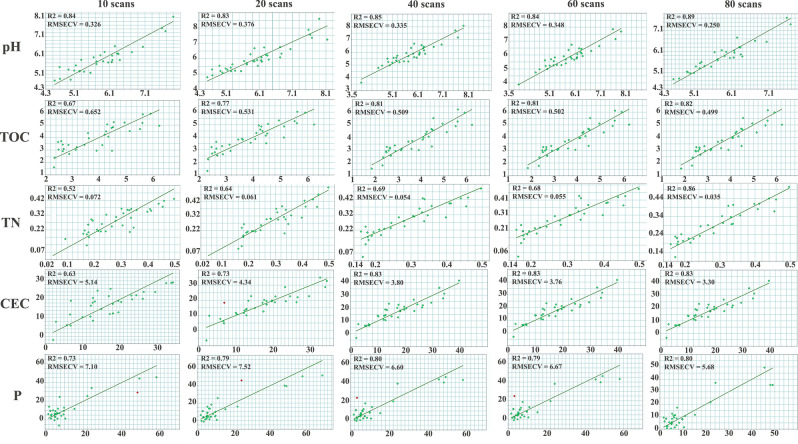
PLSR models of the five properties of interest, e.g., soil pH, TOC, TN, CEC and Olsen P depending on the scan number when recording the forty FTIR spectra.

Moreover, the improvements of the PLSR models, indicated by the increasing correlation coefficients, signifies an improved linearity between the real and predicted values and a lowering of prediction errors as shown in Figs. 5 and 6. The pH models were improved from R^2^ = 0.84 and RMSECV = 0.326 with 10 scans to R^2^ = 0.89 and RMSECV = 0.250 with 80 scans; for TOC the predictive abilities of the PLS models were upgraded from R^2^ = 0.67 and RMSECV = 0.652 with 10 to R^2^ = 0.82 and RMSECV = 0.499 when working with 80 scans, and the same trend was noticed for the other properties. The improvement was even observed with samples that were considered outliers for Olsen P prediction (Fig. 7) due to the low number of scans (i.e., 10, 20, 40 and 60), whereas when working with 80 scans these samples participated in improving the calibration. These results explained the refinement of the spectral data which reflect the enhancement of its stability after increasing the number of scans used for the final spectra. Further on, the high predictive quality of the PLSR models calibrated based on the improved database (R^2^ > 0.8 and low cross validation errors) was found even though the database contained only 40 samples, while according to the literature considerably larger databases are necessary for good models^44^. The calibrated PLS models led to high correlations and low errors compared to similar models built on the basis of big databases, e.g., Sila et al.^23^ with 1904 soil samples, Seybold et al.^42^ with about 80,000 spectra, and Baldock et al.^43^ with 20495 samples. This confirms that not only the larger number but also the quality of acquisitions influence the quality of the regression models and may even mitigate the effect of using a smaller database.

## Conclusions

In this study, we have shown that the scan number setting is an essential factor for improving the quality of FTIR spectra of soil samples. It also affected the PLSR model precision (correlation coefficient and prediction error) set up based on these soil spectra, as was shown for the prediction of five essential soil characteristics, namely soil pH, TOC, TN, CEC and Olsen P.

In the first part of this work, it was shown that the standardized moment distance index can be successfully used to assess the spectra resemblance and the SMDI approach demonstrated that above 50 scans the similarity of the acquisitions had been improved considerably, and the quality upgrading continued by increasing the number of scans. Afterward, five scan settings were studied to estimate (using the PLSR modeling algorithm) important soil properties (Viz., pH, TOC, TN, CEC and Olsen P), and the prediction results were compared. The R^2^ and RMSECV were found to be important tools for assessing the predictive models’ quality, a general tendency in improving the correlation coefficient R^2^ as the number of scans increased from 10 to 80 was noticed for all the regression models, whereas an opposite trend was noted by the cross-validation error RMSECV. Which indicates that the recorded spectra’s quality (stability and repeatability) was improved by increasing the number of scans, confirming the results obtained in the first part. In addition, this is the first known research to study the effect of the number of scans as a setup when recording the FTIR spectra on predictive models’ precision for the estimation of important soil health indicators.

The final finding of the present study, as the number of scans has a remarkable effect on spectral stability, represents an important parameter to be taken into consideration in addition to the economic and rapidity factors when recording FTIR spectra of soil samples for the set-up of predictive models in soil spectroscopy.

## Supplementary Information

Supplementary Information.

## References

[1] Nelson, W. L. *et al. Soil Testing in the United States.* (US Government Printing Office, 1951).

[2] Bray, R. H. Correlation of soil tests with crop response to added fertilizers and with fertilizer requirement. in *Diagnostic Techniques for Soils and Crops* 53–86 (American Potash Institute, 1948).

[3] Fitts JW, Nelson WL (1956). The determination of lime and fertilizer requirements of soils through chemical tests. Adv. Agron..

[4] Silveira ML, Kohmann MM, Monte Rouquette J, Aiken GE (2020). Maintaining soil fertility and health for sustainable pastures. Management Strategies for Sustainable Cattle Production in Southern Pastures.

[5] Soltani I (2019). A near infrared index to assess effects of soil texture and organic carbon content on soil water content. Eur. J. Soil Sci..

[6] Barra I, Haefele SM, Sakrabani R, Kebede F (2021). Soil spectroscopy with the use of chemometrics, machine learning and pre-processing techniques in soil diagnosis: Recent advances—A review. Trends Anal. Chem..

[7] Gomez C, Lagacherie P, Coulouma G (2012). Regional predictions of eight common soil properties and their spatial structures from hyperspectral Vis-NIR data. Geoderma.

[8] Martínez-España R, Bueno-Crespo A, Soto J, Janik LJ, Soriano-Disla JM (2019). Developing an intelligent system for the prediction of soil properties with a portable mid-infrared instrument. Biosyst. Eng..

[9] Ehsani MR, Upadhyaya SK, Slaughter D, Shafii S, Pelletier M (1999). A NIR technique for rapid determination of soil mineral nitrogen. Precis. Agric..

[10] Conforti M (2015). Laboratory-based Vis-NIR spectroscopy and partial least square regression with spatially correlated errors for predicting spatial variation of soil organic matter content. CATENA.

[11] De Luca M (2011). Derivative FTIR spectroscopy for cluster analysis and classification of morocco olive oils. Food Chem..

[12] Kharbach M (2019). Fatty-acid profiling vs UV–visible fingerprints for geographical classification of Moroccan Argan oils. Food Control.

[13] Kharbach M, Marmouzi I, El M, Bouklouze A, Vander Y (2020). Recent advances in untargeted and targeted approaches applied in herbal-extracts and essential-oils fingerprinting—A review. J. Pharm. Biomed. Anal..

[14] Barra I, Mansouri A, Bousrabat M, Cherrah Y, Kharbach M, Bouklouze A (2019). Discrimination and quantification of moroccan gasoline adulteration with diesel using Fourier transform infrared spectroscopy and chemometric tools. J. AOAC Int..

[15] Barra I, Alaoui M, Cherrah Y, Kharbach M, Bouklouze A (2019). FTIR fingerprints associated to a PLS-DA model for rapid detection of smuggled non-compliant diesel marketed in Morocco. Vib. Spectrosc..

[16] Barra I (2019). Discrimination of diesel fuels marketed in Morocco using FTIR, GC-MS analysis and chemometrics methods. Talanta.

[17] Gontijo LC (2014). Quantification of soybean biodiesels in diesel blends according to ASTM E1655 using mid-infrared spectroscopy and multivariate calibration. Fuel.

[18] Aleme HG, Costa LM, Barbeira PJS (2008). Determination of gasoline origin by distillation curves and multivariate analysis. Fuel.

[19] Wenzel T (2019). Fourier-Transform Infrared Spectroscopy (FT-IR). Molecular and Atomic Spectroscopy.

[20] Mezzetti A (2002). Rapid-scan Fourier transform infrared spectroscopy shows coupling of GLu-L212 protonation and electron transfer to QB in Rhodobacter sphaeroides reaction centers. Biochim. Biophys. Acta Bioenergy.

[21] Carra JB (2019). Near-infrared spectroscopy coupled with chemometrics tools: A rapid and non-destructive alternative on soil evaluation. Commun. Soil Sci. Plant Anal..

[22] Reeves JB, Smith DB (2009). The potential of mid- and near-infrared diffuse reflectance spectroscopy for determining major- and trace-element concentrations in soils from a geochemical survey of North America. Appl. Geochem..

[23] Sila AM, Shepherd KD, Pokhariyal GP (2016). Evaluating the utility of mid-infrared spectral subspaces for predicting soil properties. Chemom. Intell. Lab. Syst..

[24] Riefolo C (2019). Investigation of soil surface organic and inorganic carbon contents in a low-intensity farming system using laboratory visible and near-infrared spectroscopy. Arch. Agron. Soil Sci..

[25] Pimstein A, Notesco G, Ben-Dor E (2011). Performance of three identical spectrometers in retrieving soil reflectance under laboratory conditions. Soil Sci. Soc. Am. J..

[26] Salas EAL, Henebry GM (2013). A new approach for the analysis of hyperspectral data: Theory and sensitivity analysis of the moment distance method. Remote Sens..

[27] Gitelson AA, Merzlyak MN (1996). Signature analysis of leaf reflectance spectra: Algorithm development for remote sensing of chlorophyll. J. Plant Physiol..

[28] Caballero B, Finglas P, Toldra F (2016). Encyclopedia of Food and Health.

[29] Kalivas JH (2010). Calibration methodologies. Compr. Chemom..

[30] Bro R, Age KS (2014). Principal component analysis. Anal. Methods.

[31] Abdi H, Williams LJ (2010). Principal component analysis. Wiley Interdiscip. Rev. Comput. Stat..

[32] Harald M, Tormod N, Kowalski BR (1984). Multivariate calibration. Mathematics and Statistics in Chemistry.

[33] Bertrand D, Vigneau E, Dominique B, Dufour E (2006). Prétraitement des données spectrales dans la spectroscopie infrarouge et ses applications analytique. La spectroscopie infrarouge et ses applications analytiques.

[34] Esbensen KH, Guyot D, Houmøller LP (2004). An Introduction to Multivariate Data Analysis and Experimental Design. Multivariate Data Analysis.

[35] Shao J (1993). Linear model selection by cross-validation. Am. Stat. Assoc..

[36] Barra I, Mansouri Alaoui M, Bousrabat M, Cherrah Y, Bouklouze A (2019). Discrimination and quantification of moroccan gasoline adulteration with diesel using Fourier transform infrared spectroscopy and chemometric tools. J. AOAC Int..

[37] Kharbach M (2017). Characterization and classification of PGI Moroccan Argan oils based on their FTIR fingerprints and chemical composition. Chemom. Intell. Lab. Syst..

[38] Du C, Zhou J (2009). Climate Change, Intercropping, Pest Control and Beneficial Microorganisms. Evaluation of Soil Fertility Using Infrared Spectroscopy—A Review.

[39] Nocita M, Sparks DL (2015). Soil spectroscopy: An alternative to wet chemistry for soil monitoring. Advances in Agronomy.

[40] Waruru BK, Shepherd KD, Ndegwa GM, Sila A, Kamoni PT (2015). Application of mid-infrared spectroscopy for rapid characterization of key soil properties for engineering land use. Soils Found..

[41] Bruckman VJ, Wriessnig K (2013). Improved soil carbonate determination by FT-IR and X-ray analysis. Environ. Chem. Lett..

[42] Seybold CA (2019). Application of mid-infrared spectroscopy in soil survey. Soil Sci. Soc. Am. J..

[43] Baldock JA, Hawke B, Sanderman J, MacDonald LM (2013). Predicting contents of carbon and its component fractions in Australian soils from diffuse reflectance mid-infrared spectra. Soil Res..

[44] Jenkins DG, Quintana-Ascencio PF (2020). A solution to minimum sample size for regressions. PLoS ONE.

