# Supplementary material for: Optimizing setup of scan number in FTIR spectroscopy using the moment distance index and PLS regression: application to soil spectroscopy
Source: Sci Rep. 2021 Jun 25;11:13358. doi: 10.1038/s41598-021-92858-w (PMC8233441; doi:10.1038/s41598-021-92858-w)
Supplement: Supplementary file 1 — Supplementary Information. [file 41598_2021_92858_MOESM1_ESM.docx]

**Sup 1.** Performance of partial least squares regression (PLSR) models for the prediction of soil pH, TOC, TN, CEC and Olsen P by varying the scans number of the soils FTIR spectra.

| Soil property  Figure of merit | Scans number | 10 | 20 | 40 | 60 | 80 |
| --- | --- | --- | --- | --- | --- | --- |
| pH | R^2^ | 0.84 | 0.83 | 0.85 | 0.84 | 0.89 |
|  | RMSECV | 0.326 | 0.376 | 0.335 | 0.348 | 0.250 |
| TOC | R^2^ | 0.67 | 0.77 | 0.81 | 0.81 | 0.82 |
|  | RMSECV | 0.652 | 0.531 | 0.509 | 0.502 | 0.499 |
| TN | R^2^ | 0.52 | 0.64 | 0.69 | 0.68 | 0.86 |
|  | RMSECV | 0.072 | 0.061 | 0.054 | 0.055 | 0.035 |
| CEC | R^2^ | 0.63 | 0.73 | 0.83 | 0.83 | 0.83 |
|  | RMSECV | 5.14 | 4.34 | 3.80 | 3.76 | 3.30 |
| Olsen P | R^2^ | 0.73 | 0.79 | 0.80 | 0.79 | 0.80 |
|  | RMSECV | 7.10 | 7.52 | 6.60 | 6.67 | 5.68 |
